# Thermomagnetic Convection of Ferrofluid in an Enclosure Channel with an Internal Magnetic Field

**DOI:** 10.3390/mi10090553

**Published:** 2019-08-21

**Authors:** Myoungwoo Lee, Youn-Jea Kim

**Affiliations:** 1 Graduate School of Mechanical Engineering, Sungkyunkwan University, Suwon 16419, Korea; 2 School of Mechanical Engineering, Sungkyunkwan University, Suwon 16419, Korea

**Keywords:** ferrofluid, magnetic nanoparticle, magnetophoretic (MAP) force, finite element method (FEM)

## Abstract

Ferrofluid is a colloidal liquid in which magnetic nanoparticles such as Fe_3_O_4_ are dispersed in a nonconductive solution, and the average diameter of the nanoparticles is 10 nm. When a magnetic field is applied, the ferrofluid generates magnetization, which changes the physical properties of the fluid itself. In this study, characteristics of the thermomagnetic convection of ferrofluid (Fe_3_O_4_) by the permanent magnet in the enclosure channel were studied. To effectively mix the ferrofluid (Fe_3_O_4_) and disturb the boundary layer, the heat dissipation of the heat source depending on the strength of the magnetic field and the shape of the enclosure channel was numerically studied. In particular, four different enclosure channels were considered: Square, separated square, circle, and separated circle. The hot temperature was set at the center of the enclosure channel. The ferrofluid was affected by the permanent magnet in the center of the channel. The magnetic field strength in the region close to the permanent magnet was enhanced. The magnetophoretic (MAP) force increased with increasing magnetic field strength. The MAP force generated a vortex in the enclosure channel, disturbing the thermal boundary. The vortex occurs differently, depending on the shape of the enclosure channel and affects the thermomagnetic convection. The temperature and velocity fields for thermomagnetic convection were described and the convective heat flux was calculated and compared. Results show that when the magnetic field strength was 4000 kA/m and the shape of the enclosure channel was a circle, the maximum convective heat flux of 4.86 × 10^5^ W/m^2^ was obtained.

## 1. Introduction

With advances in technology, electronic devices are getting smaller and the amount of power used is increasing. The increased power generates heat in the electronics, which reduces the performance of the devices. For this reason, the importance of thermal management in the industrial field is emphasized. Recently, ferrofluid has been highlighted with cooling technology. It has higher thermal conductivity than conventional heat transfer fluid and has the advantage of controlling the fluid by using the magnetic field. Ferrofluid is a material that contains nanoparticles with nonconductive liquid (water, oil, etc.) as a solvent. The nanoparticles in the ferrofluid are Fe_3_O_4_, Al_2_O_3,_ FePt, CoPt, etc. and the average diameter of the particles is 10 nm. The ferrofluid is entirely superparamagnetic, so that the flow of ferrofluid can be controlled by the magnetic field. A magneto-hydrodynamic (MHD) method using a magnetic field is used primarily for flow control of ferrofluid, in which a magnetic force moves magnetic particles when a magnetic field is externally applied to a ferrofluid [1,2]. The flow of ferrofluid can be controlled by magnetophoresis and the temperature gradient changes, depending on the relationship between the intensity of the applied magnetic field and the flow of the ferrofluid. For these reasons, many researchers of engineering fields proposed new design parameters to various applications using fluid element [3,4,5]. Blennerhassett et al. [6] found that the ratio of convective and conduction heat transfer can be up to 10% greater than in the absence of a magnetic field through nonlinear analysis. Strek et al. [7] numerically studied the effect of a magnetic dipole located under a 2D channel on the ferrofluid in the channel. They observed that ferrofluid flows in the direction of the magnetic field at cold temperature. Aminfar et al. [8] numerically investigated the hydro-thermal characteristics of ferrofluid (water and Fe_3_O_4_) exposed to a non-uniform magnetic field, caused by the current passing through a wire placed in parallel under the duct. It was shown that heat transfer is improved by the increase of Nusselt number and friction factor when the magnetic field is applied. Song et al. [9] numerically studied the effects of permanent magnet position and intensity on thermomagnetic convection of oxygen in a rectangular enclosure. The thermomagnetic convection was largely influenced by the relative position of the permanent magnet. Tangthieng et al. [10] studied finite element simulations of heat transfer to a ferrofluid in a box in the presence of a magnetic field. Simulations showed improved heat transfer due to magnetic field gradient. Yamaguchi et al. [11] conducted experiments and numerical studies on the natural convection of ferrofluids in a 2D cell with an aspect ratio of 1. Vertically imposed magnetic fields have unstable effects and have shown that the flow is different from a state without magnetic fields. Furthermore, it has been shown that the magnetic field causes transition flow to improve heat transfer. Ghasemi et al. [12] studied the natural convection of enclosures filled with water–Al_2_O_3_ nanoparticles affected by magnetic fields. It was shown that the heat transfer rate was increased with increasing Rayleigh number but decreased with increasing Hartmann number. Jiang et al. [13] investigated numerically the thermomagnetic convection of air in a 2D square enclosure under the magnetic field on four sides. They presented the flow and temperature distributions for the thermomagnetic convection of air. The local and average Nusselt numbers on the walls were calculated and compared. The results showed that the magnetic field strength, Darcy number, and Rayleigh number affect the heat transfer in the square enclosure. Szabo et al. [14] experimentally analyzed thermodynamic convection using infrared thermography and numerically analyzed it with the finite element model. The temperature dependence of the Kelvin body force was explained by relating the heat flux and temperature profile.

Many researchers studied the thermomagnetic convection characteristics of ferrofluid in a square enclosure when applied to an external magnetic field. In this study, the thermomagnetic convection characteristic of ferrofluid (Fe_3_O_4_), depending on the enclosure channel shape (square, separated square, circle, separated circle) with the internal magnetic field, was studied using COMSOL Multiphysics 5.3a (COMSOL Inc, Burlington, MA, USA). Temperature and velocity distributions were graphically depicted with various geometrical configurations and operating conditions.

## 2. Governing Equations

Since there is no electromagnetic free current, ferrofluid can be assumed to be a nonconductor. It was assumed that there is no magnetic field effect due to the temperature gradient. Therefore, the Maxwell–Amperes law in the magnetostatics condition can be expressed as follows:

(1) $$\nabla \cdot \vec{B}=0$$

(2) $$\nabla \times \vec{H}=0$$

where $\vec{B}\;$ is the magnetic flux density vector and $\vec{H}$ is the magnetic field vector.

(3) $$\vec{H}=\frac{1}{\mu_{0}\left(1+\chi_{m}\right)}\vec{B}$$

where $\chi_{m}\;$ is the magnetic susceptibility and $\mu_{0}\;$ is the magnetic permeability in vacuum.

The viscous torque of nanoparticles can be ignored. Ferrofluids need not consider magneto- dissipation [15]. To describe the flow of ferrofluid, the following governing equations are used:

Continuity:

(4) $$\frac{\partial \rho }{\partial t}+\nabla \cdot \left(\rho \vec{v}\right)=0$$

Momentum:

(5) $$\rho \left(\frac{\partial \vec{v}}{\partial t}+\vec{v}\cdot \nabla \vec{v}\right)=-\nabla p+\nabla \cdot \tau_{ij}+\beta \rho g\left(T-T_{0}\right)\hat{k}+\left(\vec{M}\cdot \nabla \right)\vec{B}$$

Energy:

(6) $$\rho C_{p}\left(\frac{\partial T}{\partial t}+\vec{v}\cdot \nabla T\right)=k\nabla^{2}T+\eta \Phi -\mu_{0}T\frac{\partial \vec{M}}{\partial T}\left(\left(\vec{v}\cdot \nabla \right)\vec{H}\right)$$

where ρ is the density of ferrofluid, $\vec{v}$ is the velocity vector, *p* is the pressure, T is the temperature of ferrofluid, $\vec{\tau_{ij}}$ is the viscous stress tensor, $\hat{k}$ is the unit vector of gravity force, β is the thermal expansion coefficient of the ferrofluid, k is the thermal conductivity, η is the viscosity of ferrofluid, and ηΦ is the viscous dissipation term in the energy equation.

The magnetic induction vector can be expressed as follows:

(7) $$\vec{B}=\mu_{0}\left(1+\chi_{m}\right)\vec{H}$$

The variation of the total magnetic susceptibility $\chi_{m}$ is treated as being dependent on temperature [16].

(8) $$\chi_{m}=\chi_{m}\left(T\right)=\frac{\chi_{m,0}}{1+\beta \left(T-T_{0}\right)}$$

## 3. Numerical Analysis

**Figure 1:**
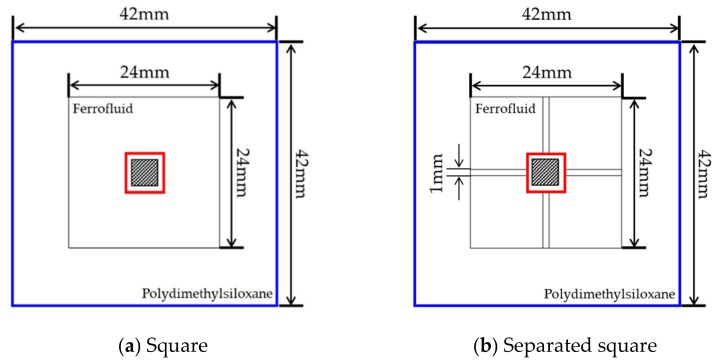

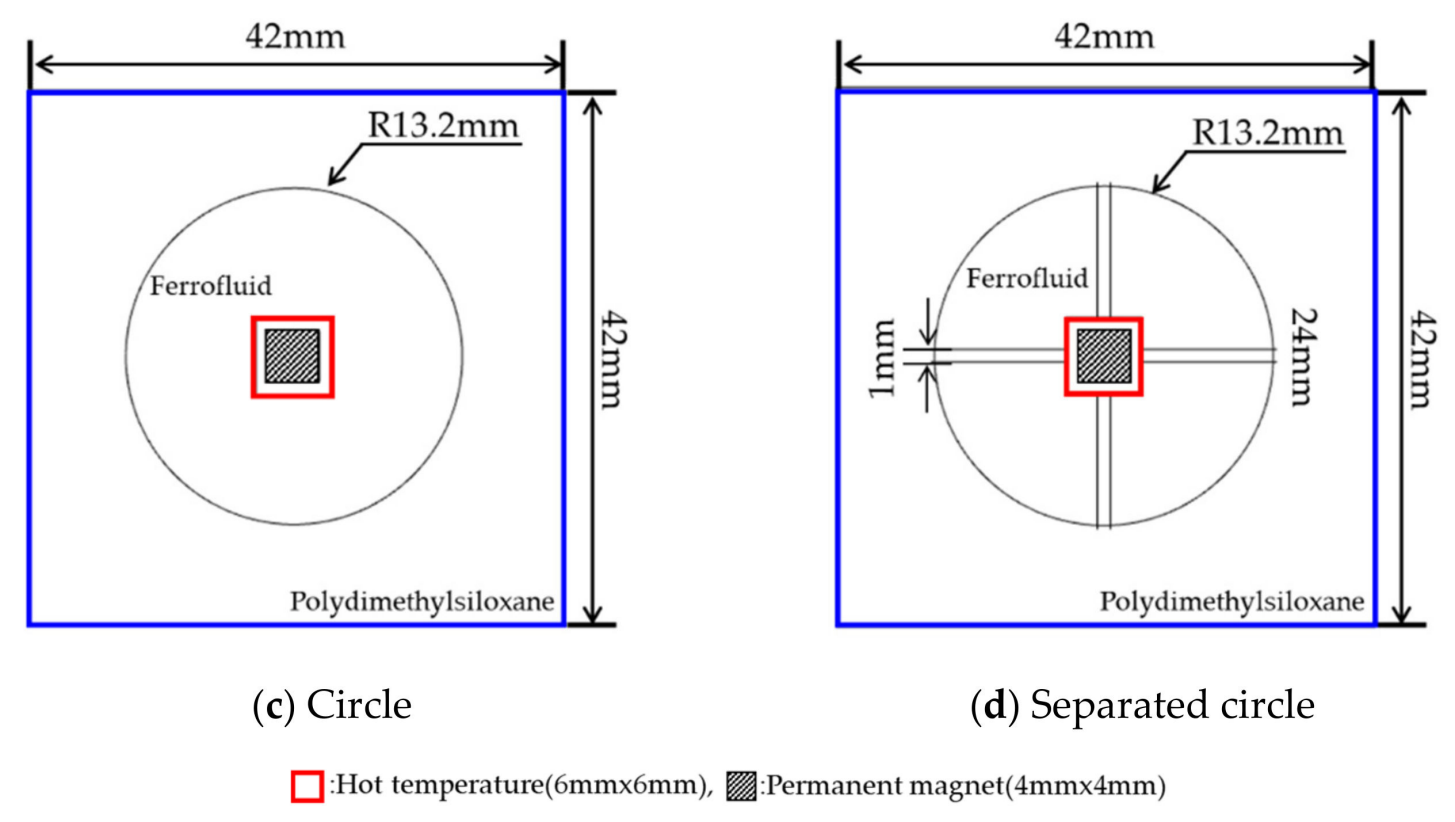
Configurations for numerical analysis.

**Figure 2:**
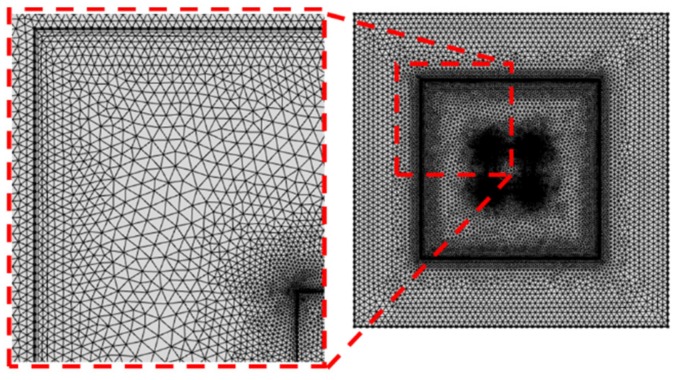
Schematic of the grid systems.

**Figure 3:**
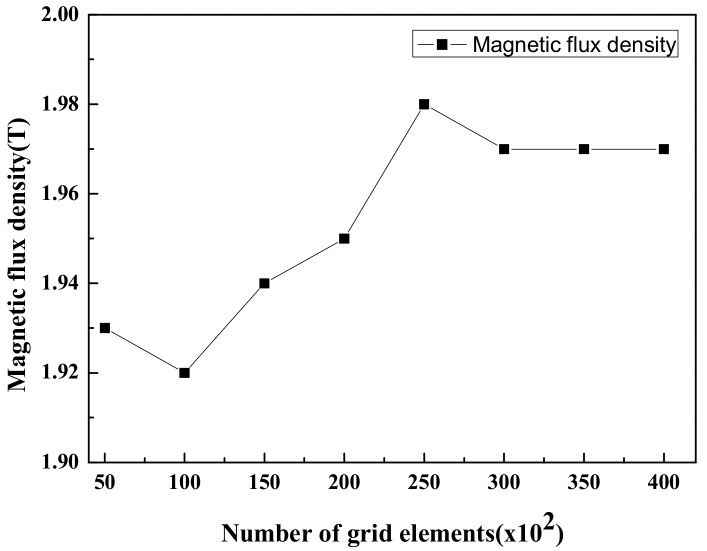
Grid dependency test.

The numerical models of this study are shown in Figure 1. The channels consisted of squares and circles with the same area. The size of the enclosure channel was set based on the conventional Central Processing Unit (CPU) chip. The polydimethylsiloxane (PDMS) was used as the material of the body and ferrofluid (Fe_3_O_4_) was used as the working fluid. The hot temperature (T_h_) in the center of the channel was 313.15 K. To apply a magnetic field, a permanent magnet was placed at the center of the channel. The magnetic field strength was changed from *H* = 1000 to 4000 kA/m to analyze the effect of the magnetic field on the ferrofluid in the enclosure channel. The grid applied to the channel is shown in Figure 2. The grid system was set up with dense free triangular for the whole model and an inflation layer was set in the vicinity of wall to check the change according to the channel. Grid dependency was tested by the magnetic flux density in the center of the channel. All cases set the mesh to 3 × 10^4^ (see Figure 3). Detailed boundary conditions are given in Table 1 and Table 2.

## 4. Results & Discussion

**Figure 4:**
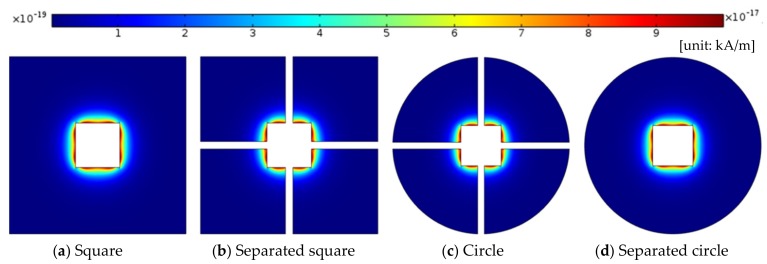
Magnetophoretic force distribution with the same magnetic field strength, *H* = 3000 (kA/m).

The applied magnetic field has a different magnetic energy distribution, depending on the shape of the channel. This affects the heat transfer characteristics of the ferrofluid in the channel. To verify the heat transfer characteristics of the ferrofluid in the channel, the influence of the magnetophoretic force due to the magnetic field was analyzed and the temperature distribution was analyzed. When the magnetic field strength is 2000 kA/m, the magnetophoretic force distributions according to the geometry are shown in Figure 4. The MAP force can be expressed as follows [17]:

(9) $${\vec{F}}_{\mathrm{MAP}}=\left(\mu_{m}M_{eff}\cdot \nabla \right)\vec{H}$$

(10) $$F_{\mathrm{MAP}}=2\pi r_{p}^{3}\mu_{m}\mathrm{ReK}\left(\mu_{p},\mu_{m}\right)\nabla H^{2}$$

where $M_{eff}$ is the magnetic dipole moment by external force, $r_{p}$ is the radius of magnetic nanoparticle, and $ReK\left(\mu_{p},\mu_{m}\right)$ is the Clausius–Mossoti factor using magnetic permeability.

**Figure 5:**
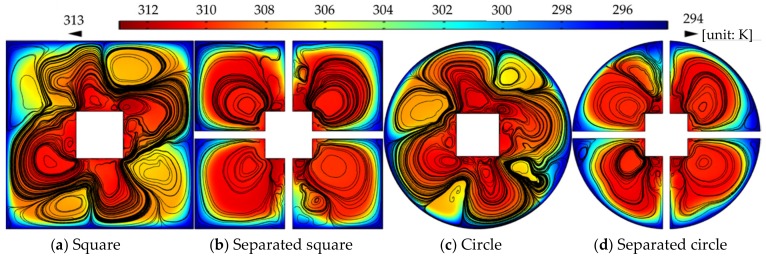
Streamlines and temperature distributions with the same magnetic field strength, *H* = 2000 (kA/m).

**Figure 6:**
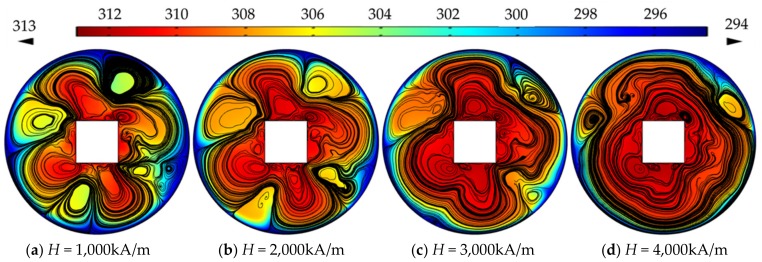
Streamlines and temperature distribution in a circle channel with the different magnetic field strength.

**Figure 7:**
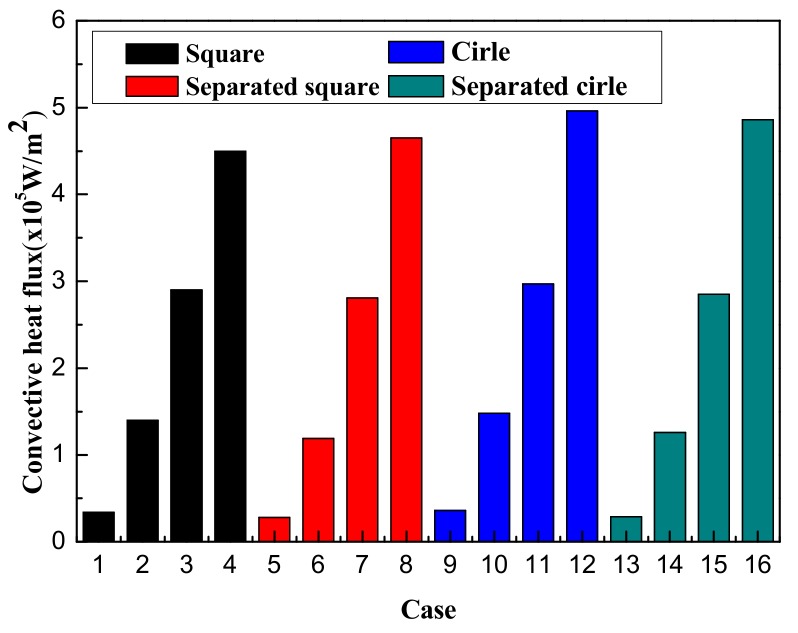
Convective heat flux in all cases.

**Figure 8:**
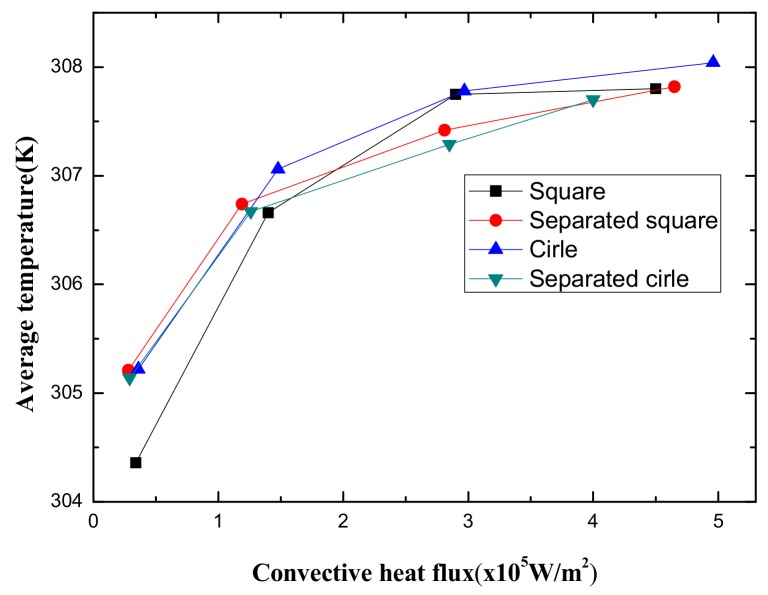
Average temperature due to convective heat flux in all cases.

The MAP force represented the behavior of the nanoparticle, due to its interaction with the magnetic field gradient. The MAP force decreased as the distance from the permanent magnet (center of the channel) increased and depended on the shape of the enclosure channel. When the magnetic field strength is *H* = 2000 kA/m, the streamlines and temperature distributions, according to the geometry, are shown in Figure 5. The temperature distribution was consistent with the streamline. The vortex was responsible for spreading the heat from the center outside. The non-separated shape showed a high temperature in a particular area and the vortex influenced the edge of the enclosure channel when the shape of the channel was circular. As the magnetic field intensity increased, a large vortex was generated in a circular channel, and the central heat shifted to the outside (see Figure 6). Figure 7 shows the convective heat flux for all cases. The heat in the center of the enclosure channel was spread by the MAP force. Therefore, the convective heat flux increased as the magnetic field strength increased. The highest convective heat flux was obtained when the channel shape was a circle. The average temperature of the channel according to the convective heat flux is shown in Figure 8. As the convective heat flux increased, the average temperature increased. However, when the convective heat flux exceeded 4 W/m^2^, the increasing slope of the average temperature value tended to decrease.

## 5. Conclusions

In this study, the thermomagnetic convection of the ferrofluid depending on the internal magnetic field was studied numerically by changing the shape of the enclosure channel. When the magnetic field was applied through the permanent magnet, the MAP force was found to occur strongly near the permanent magnet. The flow distribution of the ferrofluid formed a vortex field around the position where the MAP force was strongly generated. The MAP force plays an important role in promoting heat transfer by forming the recirculation region. The temperature distribution corresponds with the formed vortex. As the MAP force increased, the size of the vortex increased. According to the shape of the enclosure channel, a different vortex occurred in the same MAP force. Convective heat flux was larger in the circle- than in the square-shaped channel in the same area and external condition. When the magnetic field strength was 4000 kA/m and the shape of the enclosure channel was a circle, the maximum convective heat flux of 4.86 × 10^5^ W/m^2^ was obtained. Through study, it was confirmed that the enclosure channel shape and magnetic field affect the thermodynamic convection characteristics of the ferrofluid. It is possible to embody the effective heat transfer performance through the magnetic field and channel design.

## Figures and Tables

**Table 1:** Physical properties of the ferrofluid (EFH-1).

Density	1221 kg/m³
Relative permeability of ferrofluid	1.01
Relative permeability of magnetite (Fe_3_O_4_)	16
Magnetic susceptibility	1.552
Dynamic viscosity	0.00727 Pa∙s
Thermal conductivity	0.19W/m∙K
Heat capacity at static pressure	1840 J/kg∙K
Thermal expansion coefficient	8.6 × 10^−4^ (1/K)

**Table 2:** Boundary conditions applied in this study.

Working fluid	Ferrofluid
Geometric configurations	Square, Separated square, Circle, Separated circle
Magnetic field strength, *H*	1000–4000 (kA/m)
Directions of magnetic energy	Horizontal
Initial temperature	293.15 K
Hot temperature (Heat source), *T_h_*	313.15 K
Wall conditions of fluid domain	No-slip condition
